# Use of a High Resolution Melting (HRM) Assay to Compare *Gag*, *Pol*, and *Env* Diversity in Adults with Different Stages of HIV Infection

**DOI:** 10.1371/journal.pone.0027211

**Published:** 2011-11-02

**Authors:** Matthew M. Cousins, Oliver Laeyendecker, Geetha Beauchamp, Ronald Brookmeyer, William I. Towler, Sarah E. Hudelson, Leila Khaki, Beryl Koblin, Margaret Chesney, Richard D. Moore, Gabor D. Kelen, Thomas Coates, Connie Celum, Susan P. Buchbinder, George R. Seage, Thomas C. Quinn, Deborah Donnell, Susan H. Eshleman

**Affiliations:** 1 Department of Pathology, Johns Hopkins University School of Medicine, Baltimore, Maryland, United States of America; 2 Department of Medicine, Johns Hopkins University School of Medicine, Baltimore, Maryland, United States of America; 3 Laboratory of Immunoregulation, National Institute of Allergy and Infectious Diseases, National Institutes of Health, Baltimore, Maryland, United States of America; 4 Statistical Center for HIV/AIDS Research and Prevention, Fred Hutchinson Cancer Research Center, Seattle, Washington, United States of America; 5 Department of Biostatistics, University of California Los Angeles, Los Angeles, California, United States of America; 6 Laboratory of Infectious Disease Prevention, New York Blood Center, New York, New York, United States of America; 7 University of California San Francisco, San Francisco, California, United States of America; 8 Department of Emergency Medicine, Johns Hopkins University School of Medicine, Baltimore, Maryland, United States of America; 9 Program in Global Health, University of California Los Angeles, Los Angeles, California, United States of America; 10 Department of Medicine, University of Washington, Seattle, Washington, United States of America; 11 San Francisco Department of Public Health, San Francisco, California, United States of America; 12 Department of Epidemiology, Harvard School of Public Health, Boston, Massachusetts, United States of America; National AIDS Research Institute, India

## Abstract

**Background:**

Cross-sectional assessment of HIV incidence relies on laboratory methods to discriminate between recent and non-recent HIV infection. Because HIV diversifies over time in infected individuals, HIV diversity may serve as a biomarker for assessing HIV incidence. We used a high resolution melting (HRM) diversity assay to compare HIV diversity in adults with different stages of HIV infection. This assay provides a single numeric HRM score that reflects the level of genetic diversity of HIV in a sample from an infected individual.

**Methods:**

HIV diversity was measured in 203 adults: 20 with acute HIV infection (RNA positive, antibody negative), 116 with recent HIV infection (tested a median of 189 days after a previous negative HIV test, range 14–540 days), and 67 with non-recent HIV infection (HIV infected >2 years). HRM scores were generated for two regions in *gag*, one region in *pol*, and three regions in *env*.

**Results:**

Median HRM scores were higher in non-recent infection than in recent infection for all six regions tested. In multivariate models, higher HRM scores in three of the six regions were independently associated with non-recent HIV infection.

**Conclusions:**

The HRM diversity assay provides a simple, scalable method for measuring HIV diversity. HRM scores, which reflect the genetic diversity in a viral population, may be useful biomarkers for evaluation of HIV incidence, particularly if multiple regions of the HIV genome are examined.

## Introduction

Accurate methods for measuring HIV incidence using cross-sectional samples are important for monitoring the HIV epidemic and assessing the efficacy of interventions for HIV prevention [1]. Most cross-sectional HIV incidence studies have been performed using serologic assays, such as the BED capture immunoassay [2]. Serologic HIV incidence assays are based on the premise that the antibody response to HIV matures over time. Unfortunately, some individuals never attain a mature anti-HIV antibody response, and individuals with non-recent HIV infection may be misclassified as recently infected if the antibody response to HIV is blunted by viral suppression or advanced HIV disease [3], [4].

Because HIV generally diversifies over time in infected individuals, HIV diversity may serve as a biomarker for assessing HIV incidence [5]. Most studies of HIV diversity have used sequence-based methods to analyze individual HIV variants in infected individuals [6], [7], [8]. Those studies demonstrate that HIV infection is usually initiated by one or a small number of founder virions [9]. Over time, rapid viral replication, frequent mutation, and frequent recombination events generate large numbers of distinct viral variants [9], [10]. Immune responses to infection, antiretroviral therapy (ART), and other selective pressures drive the diversification and evolution of the viral population [9], [11]. Previous reports suggest that *env* sequences are usually homogeneous early in infection [12], [13], [14], with higher levels of diversity accompanying higher multiplicity of infection [12], [13]. After HIV infection is established, *env* diversity usually increases over time and stabilizes or declines in advanced stages of HIV disease [7], [14], [15]. Genetic diversification in *env* and *gag* may be concordant or discordant over the course of infection [16], [17]. Differences in viral diversification in these two regions may reflect different selective pressures targeting *env* and *gag* proteins [16], [18].

While sequencing-based studies of HIV diversity have been informative, the cost and effort needed to sequence viral variants (e.g., by cloning, single genome sequencing, or parallel sequencing) make those methods impractical for analysis of HIV incidence in larger cohorts or surveillance studies, which may require analysis of hundreds or thousands of samples. Recent reports have found that the frequency of ambiguous nucleotide calls in population sequencing data may reflect HIV diversity [19], [20]. This approach may be useful for assessing HIV diversity using existing sequence databases generated for surveillance of HIV drug resistance. However, patterns of HIV diversity can vary from one genomic region to another, and genetic bottlenecking may occur in some regions during the course of HIV infection. Therefore, discrimination between recent and non-recent HIV infection may require analysis of diversity in more than one region of the HIV genome. For such an approach to be practical for HIV incidence applications, simpler methods are needed for HIV diversity analysis. Heteroduplex mobility assays can be used to analyze HIV diversity without sequencing [14]. In those assays, genetic diversity is quantified by analyzing the mobility pattern of amplified DNA in a gel. Unfortunately, the requirement for gel electrophoresis increases the time and effort needed for analysis and makes heteroduplex mobility assays difficult to scale up for high-throughput analysis.

We recently developed a rapid assay for HIV diversity based on high resolution melting (HRM) technology [21]. Assays based on HRM of DNA duplexes have been used to detect mutations associated with cancer and genetic diseases; HRM technology is also being developed for analysis of specific mutations in bacterial, viral, and parasitic pathogens [22]. We adapted HRM technology to quantify genetic diversity in HIV [21], [23]. The HRM diversity assay is performed in a 96-well plate format, and each melting procedure takes only a few minutes. The HRM diversity assay provides a single numeric HRM score that reflects the level of HIV diversity in a specific region of the HIV genome, simplifying data analysis. Calculation of the HRM score is straightforward and can be automated using the electronic output of the melting instrument. The HRM diversity assay is highly reproducible, and HRM scores are significantly associated with sequence-based diversity measures such as genetic diversity, genetic complexity, and Shannon entropy [21], [23]. In this report, the HRM diversity assay was used to compare, *gag, pol*, and *env* diversity in samples from 203 adults with different stages of HIV infection. These data suggest that the HRM diversity assay may be useful for analysis of HIV incidence.

## Methods

### Human subjects (Ethics Statement)

The EXPLORE, HIVNET 001, Johns Hopkins HIV Clinical Cohort (JHHCC), and Johns Hopkins Hospital Emergency Department (JHH ED) studies were conducted according to the ethical standards set forth by the institutional review boards of the participating institutions and the Helsinki Declaration of the World Medical Association; participants provided written, informed consent [24], [25], [26], [27]. The work described in this report involved analysis of stored samples and data from those studies. No participants were recruited or followed in the course of this work. The work described in this report was approved by the Internal Review Board at the Johns Hopkins University School of Medicine.

### Samples used for analysis

Samples were collected from adults in the United States. Acute samples (HIV RNA positive, HIV antibody negative [28], Feibig stage I or II [29], n = 20) and recent samples (collected near the time of HIV seroconversion, likely Feibig stage VI [29], n = 102) were obtained from men who have sex with men (MSM) in the EXPLORE study [24] (1999-2001, median age: 31 years, range 19-56 years, 66% Caucasian). Additional recent samples (collected near the time of HIV seroconversion, likely Feibig stage VI [29], n = 14) were obtained from the HIVNET 001 study [25] (1995–1997) from ten heterosexual intravenous drug users (IDUs, six women and four men) and four heterosexual women who were not IDUs. The median time between collection of recent samples and the last negative HIV test was 189 days (range 14–540) for the EXPLORE study and 165 days (range: 49–216) for the HIVNET 001 study.

Samples from adults with non-recent HIV infection (infected >2 years) were obtained from two sources: (1) The Johns Hopkins HIV Clinical Cohort (JHHCC) study of HIV-infected patients in Baltimore (2002–2008, n = 56) [26], and (2) Johns Hopkins Hospital Emergency Department (JHH ED) HIV serosurvey (2001 and 2003, n = 11) [27]. Characteristics of the non-recent group (n = 67, JHHCC and JHH ED) were: 72% men, 36% Caucasian (53 had data for gender or race), median age: 36 years (range: 21–53 years; 60 had data for age), median log_10_ HIV viral load: 5.2 log_10_ copies/ml (range: 3.6–5.9 log_10_ copies/ml, 55 had data for viral load), and median CD4 cell count: 43 cells/mm^3^ (range: 1–388 cells/mm^3^, 57 had data for CD4 cell count).

### Preparation of DNA for HRM analysis

HIV RNA was extracted from plasma or serum using the ViroSeq HIV Genotyping System (Celera, Alameda, CA). HIV DNA used to analyze *gag* and *pol* was prepared using the ViroSeq system. HIV DNA used to analyze *env* was prepared using the Qiagen OneStep RT-PCR Kit (QIAGEN Inc., Valencia, CA; forward primer: JH35F (5′-TGARGGACAATTGGAGAARTGA-3′); reverse primer JH38R (5′-GGTGARTATCCCTKCCTAAC-3′) [30], [31]). PCR products were purified using ExoSAP-IT (United States Biochemical Corporation, Cleveland, OH) and were diluted to approximately 0.5 ng/µl for HRM analysis.

### Preparation of plasmid controls

DNA (*gag*-*pol* and *env* amplicons) amplified from five recent samples (EXPLORE study) was cloned into the vector, pCR®2.1-TOPO (TOPO TA Cloning® Kit, Invitrogen, Carlsbad, CA). Plasmids were diluted to approximately 5 ng/µl for HRM analysis.

### HRM diversity analysis

**Figure 1:**
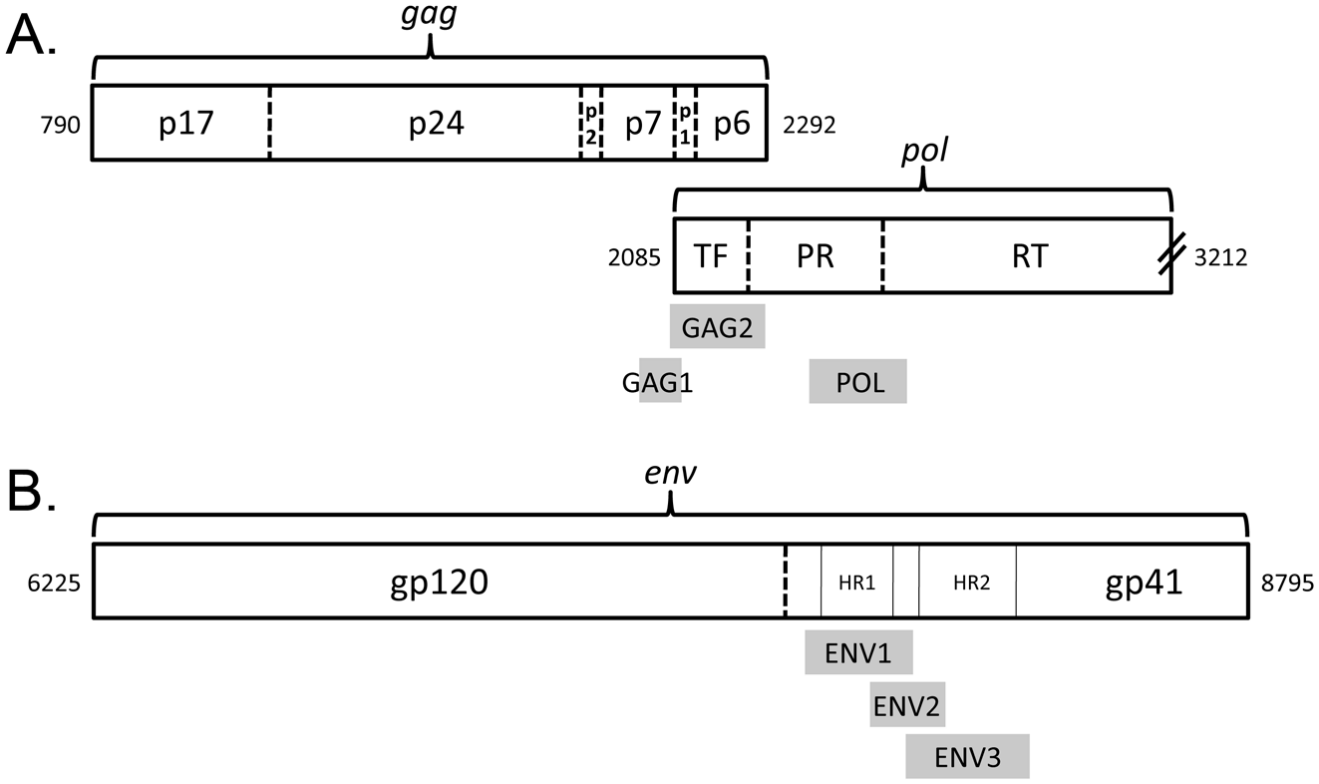
Regions of the HIV genome analyzed using the HRM diversity assay. The relevant regions of the HIV genome are shown (adapted from [39]). Numbers at the ends of each genomic segment correspond to coordinates in HXB2 (Genbank accession number: K03455). The amplicons used for HRM diversity analysis (regions analyzed) are indicated by shaded boxes. (A) *Gag* and *pol* amplicons: The GAG1 amplicon includes a portion of the coding regions for *gag* p7 and *gag* p1. The GAG2 amplicon includes the coding regions for *gag* p1 and *gag* p6 and extends into the coding region for HIV protease (PR); this amplicon also corresponds to the transframe (TF) protein. The POL amplicon spans the junction between the coding regions of HIV protease and HIV reverse transcriptase (RT). (B) *Env* amplicons: The ENV1 amplicon includes the coding region for heptad repeat 1 (HR1) of gp41, as well as portions of the coding regions to either side of HR1. The ENV3 amplicon includes the coding region for heptad repeat 2 (HR2), as well as portions of the coding regions to either side of HR2. The ENV2 amplicon includes the coding region for immunodominant region (IDR) cluster I of gp41, as well as portions of the coding regions for HR1 and HR2 [33].

The HRM diversity assay was performed as previously described [21]. Six regions of the HIV genome were amplified in the presence of LCGreen® Plus dye (Idaho Technology Inc., Salt Lake City, UT, Table 1, Figure 1). The amplicons were melted using the LightScanner Instrument (Model HR 96, Idaho Technology Inc., Salt Lake City, UT), and release of the dye was quantified as a function of temperature (melting range for *gag* and *pol* amplicons: 68–98°C with a 65°C hold; melting range for *env* amplicons: 60–98°C with a 57°C hold). Melting curves were used to determine HRM scores, as described previously [21], using a 15°C window size. Samples were analyzed in duplicate and the results were averaged; if the difference in the duplicate HRM scores was >0.5, the data were rejected, and the samples were reanalyzed.

**Table 1:** Regions of the HIV genome analyzed using the high resolution melting (HRM) diversity assay.

Region analyzed^a^	Corresponding region in HXB2^a^	Sequences of primers used to produce amplicons for HRM diversity analysis^b^	Amplicon size (bp)
GAG1	1998 – 2097	Forward: 5′- AAATTGCAGGGCCCCTAGGAA	100
		Reverse: 5′- TTTCCCTAAAAAATTAGCCTGTCT	
GAG2	2068 – 2278	Forward: 5′- ACTGAGAGACAGGCTAATTTTTTAG	211
		Reverse: 5′- GGTCGTTGCCAAAGAGTGATTTG	
POL	2373 – 2597	Forward: 5′- AAATGGAAACCAAAAATGATAG	225
		Reverse: 5′- CATTCCTGGCTTTAATTTTACTG	
ENV1	7798 – 8036	Forward: 5′- CAGCAGGWAGCACKATGGG	239
		Reverse: 5′- GCARATGWGYTTTCCAGAGCADCC	
ENV2	7950 – 8119	Forward: 5′- CTYCAGRCAAGARTCYTGGC	170
		Reverse: 5′- TCCCAYTSCAKCCARGTC	
ENV3	8016 – 8299	Forward: 5′- TGCTCTGGAAARCWCATYTGC	284
		Reverse: 5′- AARCCTCCTACTATCATTATRA	

a See Figure 1.

b Mixed nucleotides were present at some positions (W: A/T; K: G/T; R: A/G; Y: C/T; D: A/G/T; S: G/C).

### Statistical methods

The Wilcoxon rank sum test was used to compare HRM scores from different sample sets (e.g., non-recent vs. recent) for each region. Outlier values were defined as greater than the third quartile + (1.5 x interquartile range [IQR]) or less than the first quartile – (1.5 x IQR). Extreme values were defined as values greater than the third quartile + (3 x IQR) or less than the first quartile – (3 x IQR). Logistic regression was used to assess HRM scores for recent and non-recent infection for all six regions both separately and jointly. The Wilcoxon rank sum test was used to compare HRM scores for adults in different subgroups (e.g., with and without ART). Analyses were performed using SAS software version 9.2 (Cary, North Carolina).

## Results

**Figure 2:**
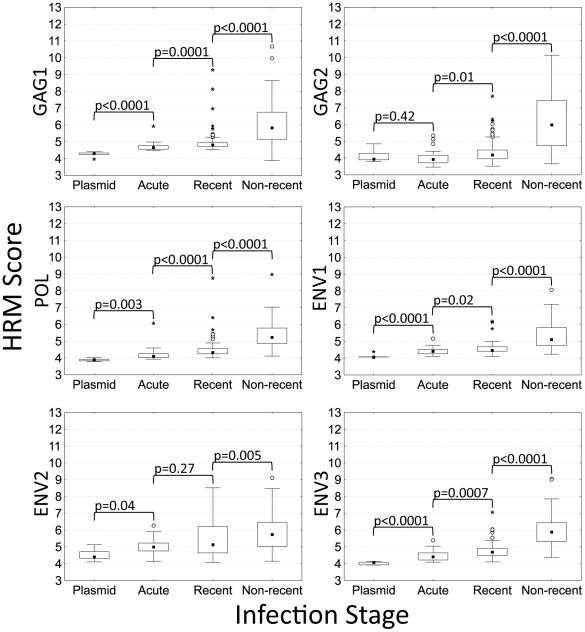
HRM scores for plasmid controls and samples from adults with different stages of HIV disease. The box and whisker plots show the distribution of HRM scores for six regions in the HIV genome in control plasmids (n = 5; Subtype B) and in adults with acute (n = 20), recent (n = 102), and non-recent (n = 67) HIV infection (see text). For each column, the median (closed square), interquartile range (IQR, box), lower inner fence (first quartile [Q1] – [1.5 X IQR]) and upper inner fence (third quartile [Q3] + [1.5 X IQR], whiskers), outliers (greater than [Q3] + [1.5 x IQR], open circle) and extremes (greater than [Q3] + [3 x IQR], asterisk) are shown.

The HRM diversity assay was used to analyze six regions in the HIV genome: two regions in HIV *gag* (GAG1 and GAG2), one region in HIV *pol* (POL), and three regions in HIV *env* (ENV1, ENV2, and ENV3; Figure 1, Table 1). HRM scores for each region were all less than 5.2 for control plasmids (Figure 2). Region-specific differences in HRM scores for the plasmids most likely reflect differences in the length and melting domain characteristics of the amplicons [32].

The HRM diversity assay was used to analyze samples from 189 adults with different stages of HIV infection (20 with acute HIV infection [EXPLORE study], 102 with recent HIV infection [EXPLORE study], and 67 with non-recent HIV infection [JHHCC and JHU ED], see Methods). ART use and low CD4 cell count have been associated with misclassification of individuals with non-recent infection as recently infected using serologic HIV incidence assays [3], [4]. To test whether this type of misclassification would also complicate use of the HRM diversity assay for HIV incidence testing, the non-recent sample set was selected to include a high proportion of “challenge” samples; 30 (44.8%) of the 67 non-recent samples were from adults on ART and 32 (48%) of the 67 non-recent samples were from adults with advanced HIV disease (CD4 cell counts <50 cells/mm^3^).

The HRM scores obtained for adults with acute HIV infection were all low (<6.3). The median HRM scores for those samples were significantly higher than the median scores for control plasmids for all of the regions except GAG2 (Figure 2). The highest median HRM score for acute samples was obtained for the ENV2 region which includes the immunodominant region (IDR) cluster I of HIV gp41 (Figure 1) [33]. The median HRM scores obtained for adults with recent HIV infection (seroconversion samples) were significantly higher than the median scores obtained for adults with acute infection for all of the regions except ENV2 (Figure 2).

**Figure 3:**
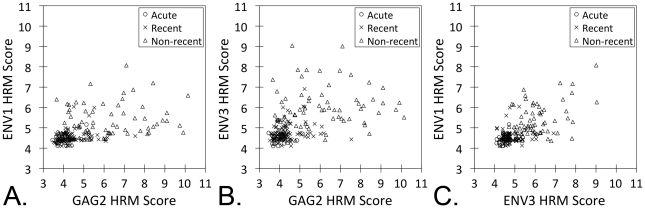
Relationship between HRM scores for the ENV1, ENV2, and GAG2 regions. Scatter plots are shown for HRM scores for adults with acute, recent, and non-recent infection: (A) ENV1 vs. GAG2, (B) ENV3 vs. GAG2, and (C) ENV1 vs. ENV3.

**Figure 4:**
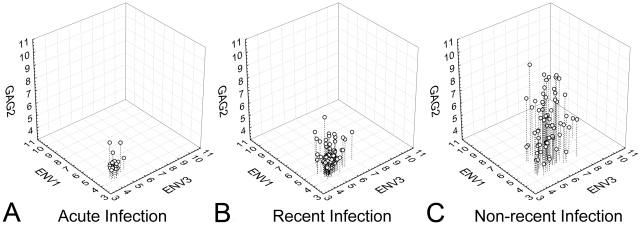
HRM diversity scores for GAG2, ENV1 and ENV3 plotted in 3 dimensions. High HRM scores for these regions were determined to be independently associated with non-recent infection. The use of data from multiple regions in tandem demonstrates that HRM scores are generally compact in acute infection (A) with a slight increase in the distribution of data in recent infection (B), and wide dispersion of the data in non-recent infection (C).

In all six regions analyzed, the median HRM scores for adults with non-recent infection (infected >2 years) were significantly higher than the median scores for adults with recent HIV infection (P = 0.005 for ENV2, P<0.0001 for the other regions, Figure 2). Higher HRM scores in each region were associated with non-recent infection in logistic regression models (compared to recent HIV infection, P<0.02 for ENV2, P<0.0001 for all other regions; Table 2). In a multivariate logistic regression model, HRM scores in three regions were independently associated with non-recent infection (GAG2: P<0.04, ENV1: P<0.004, and ENV2: P<0.0004; Table 2). Correlation plots for HRM scores in these three regions in adults with acute, recent, and non-recent infection are shown in Figure 3. In general, acute and recent infection samples had low HRM scores in all three regions, while non-recent infection samples had higher HRM scores (e.g., >6) in at least one of the three regions (Figures 3 and 4).

**Table 2:** Comparison of HRM scores from adults with recent vs. non-recent HIV infection*.

Region analyzed^a^	Univariate model		Multivariate model	
	Odds ratio (95% CI)	P value	Odds ratio^b^ (95% CI)	P value
GAG1	**0.20 (0.10–0.34)**	**<0.001**	0.60 (0.29–1.18)	0.15
GAG2	**0.27 (0.17–0.40)**	**<0.001**	**0.58 (0.32–0.95)**	**0.04**
POL	**0.10 (0.05–0.20)**	**<0.001**	0.60 (0.25–1.54)	0.33
ENV1	**0.03 (0.006–0.08)**	**<0.001**	**0.17 (0.04–0.52)**	**0.004**
ENV2	**0.72 (0.54–0.95)**	**0.02**	0.92 (0.56–1.51)	0.73
ENV3	**0.07 (0.03–0.15)**	**<0.001**	**0.20 (0.07–0.46)**	**<0.001**

*Abbreviations: HRM: high resolution melting. CI: confidence intervals. Recent samples were collected a median of 189 days after a negative HIV test (range: 14–540 days); non-recent samples were from individuals infected >2 years (see Methods). Results from adults with acute HIV infection were not included in this analysis. Statistically significant values are shown in bold text.

a See Figure 1.

b Based on multivariate logistic regression adjusting for all regions simultaneously.

HRM scores for adults with non-recent infection who had CD4 cell counts above vs. below 50 cells/mm^3^ were not significantly different for five of the six regions analyzed. For the POL region, the median HRM score was 5.6 for adults with CD4 cell counts <50 cells/mm^3^ vs. 5.1 for adults with CD4 cell counts >50 cells/mm^3^ (P = 0.006, Wilcoxon test). In Figure 2, we show the distribution of the HRM scores obtained for each group of participants. A total of 402 HRM scores were included in the analysis of participants with non-recent infection (67 samples, 6 regions each). Six (1.5%) of the 402 HRM scores were outlier or extreme values (see Methods); all six of those scores were from adults who had CD4 cell counts <50 cells/mm^3^. As shown in Figure 2, all of those outlier / extreme values are high values, indicating the presence of viral populations that had high levels of diversity in the regions analyzed. Note that all of the HRM scores (including the outlier / extreme HRM scores) were included in the analysis. The unusually high levels of viral diversity in some adults with very low CD4 cell counts enhances our ability to discriminate between adults with recent HIV infection and adults with non-recent HIV infection, including those with advanced HIV disease. There was no significant association between antiretroviral drug use and HRM score for any of the six regions (not shown).

In the analysis described above, adults with recent infection were all MSM; three (3%) of those men reported using intravenous drugs in the six months before HIV seroconversion. In contrast, the non-recent group included both men and women, most of whom likely acquired HIV infection through intravenous drug use. Some studies indicate that the number of HIV variants present very early in HIV infection varies among different risk groups [12], [13], [34], [35]. Therefore, differences in HRM scores between these two groups (recent and non-recent) may have reflected their different demographic characteristics and risk factors for HIV acquisition, rather than the duration of HIV infection. To address this possibility, we tested additional samples from women and heterosexual men, most of whom were IDUs (HIVNET 001 cohort, see Methods). In the GAG1, GAG2, POL, and ENV3 regions, HRM scores for men in the EXPLORE cohort and the adults in the HIVNET 001 cohort were not significantly different; HRM scores for two regions (ENV1 and ENV2) were slightly lower for adults in the HIVNET 001 cohort (Table 3). This indicates that the lower HRM scores observed in the recent group from EXPLORE compared to the non-recent group (from the JHHCC and JHU ED serosurvey) most likely reflect differences in HIV diversity in recent vs. non-recent HIV infection rather than differences in the demographic characteristics and risk factors of the specific cohorts tested.

**Table 3:** Comparison of HRM scores from recently infected individuals from the HIVNET 001 cohort and the EXPLORE cohort*.

Region analyzed^a^	HRM Score b		P value^c^
	HIVNET 001 (n = 14)	EXPLORE (n = 102)	
GAG1	4.8 (4.7, 4.9)	4.8 (4.7, 4.9)	0.95
GAG2	4.4 (4.1, 4.7)	4.2 (4.0, 4.5)	0.13
POL	4.3 (4.1, 4.4)	4.3 (4.3, 4.6)	0.24
ENV1	4.4 (4.3, 4.4)	4.5 (4.4, 4.7)	0.02
ENV2	4.6 (4.4, 4.8)	5.1 (4.6, 6.2)	0.01
ENV3	4.7 (4.4, 5.6)	4.7 (4.5, 4.9)	0.55

*Abbreviations: HRM: high resolution melting. CI: confidence intervals.

a See Figure 1.

b HRM scores were obtained for samples collected from adults with recent HIV infection. Samples from the HIVNET 001 cohort were obtained from women and from men who likely acquired HIV through intravenous drug use. Samples from the EXPLORE cohort were obtained from men who likely acquired HIV infection through sexual exposure with other men. The median number of days between collection of the sample used for testing and the individual's last negative HIV tests was 165 days (range: 49–216) for the HIVNET 001 cohort and 189 days (range: 14–540) for the EXPLORE cohort. Median HRM scores and the first and third quartile HRM scores (in parentheses) are shown.

c Wilcoxon Rank sum test.

## Discussion

We used a novel HRM diversity assay to compare HIV diversity in adults with different stages of HIV disease. Adults with acute HIV infection had uniformly low HRM scores (low levels of HIV diversity). However, the median HRM scores for those individuals were significantly higher than those obtained for plasmid controls for all regions except GAG2. This indicates that the HRM diversity assay can detect a low level of HIV diversity very early in HIV infection. In adults with acute HIV infection, the highest median HRM score was obtained for the ENV2 region, which contains IDR cluster I of gp41. HRM scores for all regions except for ENV2 were significantly higher in seropositive adults with recent HIV infection than in adults with acute HIV infection.

In all six regions analyzed, we found significantly higher levels of HIV diversity in adults who were infected for at least two years (non-recent group) than in adults near the time of HIV seroconversion (recent group). An important finding of this study was that viruses from individuals with non-recent infection often exhibited low diversity in at least one of the regions analyzed; in each region, there was some overlap in the HRM scores from adults with recent and non-recent infection. A similar finding was reported in a study that used a heteroduplex mobility assay to compare HIV diversity in the V3-V5 region of HIV *env* in adults with likely recent vs. likely non-recent infection (classified using a detuned enzyme immunoassay strategy) [36]. These findings and ours indicate that diversity-based measures (obtained using the HRM diversity assay or another method for viral diversity analysis) are not likely to be useful for HIV incidence analysis if they rely on analysis of a single genomic region. Our study extends the previous report [36] by comparing diversity in multiple genomic regions and by using sample sets from individuals with known recent and known non-recent infection. This expanded analysis revealed that individuals with non-recent HIV infection rarely had low diversity in all regions that we examined. Furthermore, multivariate logistic regression showed that higher HRM scores in three regions (GAG2, ENV1, and ENV3) were independently associated with non-recent HIV infection. Independent diversification in different regions of the HIV genome is likely to reflect several factors, including: (1) different selective forces act on different HIV gene products, inducing diversification of different genomic regions, and (2) the very high frequency of genetic recombination of HIV lowers genetic linkage of different subgenomic regions. Our findings suggest that HIV diversity may be a useful biomarker for HIV incidence determination, provided that multiple HIV genomic regions are analyzed. The HRM diversity assay is simpler and less expensive than many other laboratory approaches used to measure HIV diversity, and it is particularly well-suited to multi-region analysis. The HRM diversity assay can be used to measure diversity in any RNA or DNA sample, including HIV RNA and proviral DNA. For HIV incidence applications, it makes most sense to analyze the actively replicating pool (e.g., plasma HIV RNA) rather than proviral DNA, which is likely to include archived sequences from viruses that were circulating earlier in infection.

Advanced HIV disease is associated with misclassification of individuals with non-recent infection as recently infected using serologic incidence assays [3]. Our results indicate that this is not likely to confound the use of the HRM diversity assay for HIV incidence testing. In this study, almost half (48%) of the samples in the non-recent group were from individuals with CD4 cell counts <50 cells/mm^3^. In the non-recent group, HRM scores were not significantly different among adults with CD4 cell counts above vs. below 50 cells/mm^3^, and all of the unusually high HRM scores in this group (outlier and extreme values) were from adults with CD4 cell counts <50 cells/mm^3^. These data show that advanced HIV disease is not associated with misclassification using the HRM diversity assay, and suggest that the HRM diversity assay may be useful for identifying samples from adults with advanced HIV disease who were misclassified as recently infected using serologic incidence assays.

Viral suppression is also associated with misclassification of individuals with non-recent infection as recently infected using serologic incidence assays [3]. In this study, samples from adults on ART had detectable HIV RNA (to permit amplification of HIV RNA for analysis); it is not known whether those individuals were non-adherent to their treatment regimens or were failing ART. Because the HRM diversity assay uses different primer pairs for amplification of various regions of the HIV genome, and because the primers are designed to bind to relatively conserved sequences, sequence differences in HIV samples are not likely to impair binding of all of the relevant primer pairs. Therefore, amplification failure for all primer pairs is likely to indicate low viral load. We do not feel that it is necessary to screen samples for viral load prior to testing with the HRM diversity assay. If desired, a viral load assay could be used to confirm viral suppression in samples with multi-region amplification failure. We recognize that recently-infected individuals who have very low viral loads could be misclassified as non-recent if virologic suppression is used as a biomarker for non-recent infection. However, because infected individuals are not likely to have natural or ART-induced viral suppression early in infection, this type of misclassification should be infrequent and should have very little impact on HIV incidence estimates.

For samples that do amplify, we recognize that there is a potential to underestimate diversity when fewer copies of HIV RNA are used in the analysis. However, in a previous study [23], we demonstrated that results from the HRM diversity assay were not significantly affected by differences in sample volume (0.1 vs. 0.5 ml), HIV viral load (range: 2,000 to 50,000 copies/ml), or the number of HIV RNA copies used to prepare DNA templates for amplification (range: 100 to 5,000 copies of HIV RNA). Those results support the use of the HRM diversity assay for analysis of clinical samples with variable viral loads.

**Figure 5:**
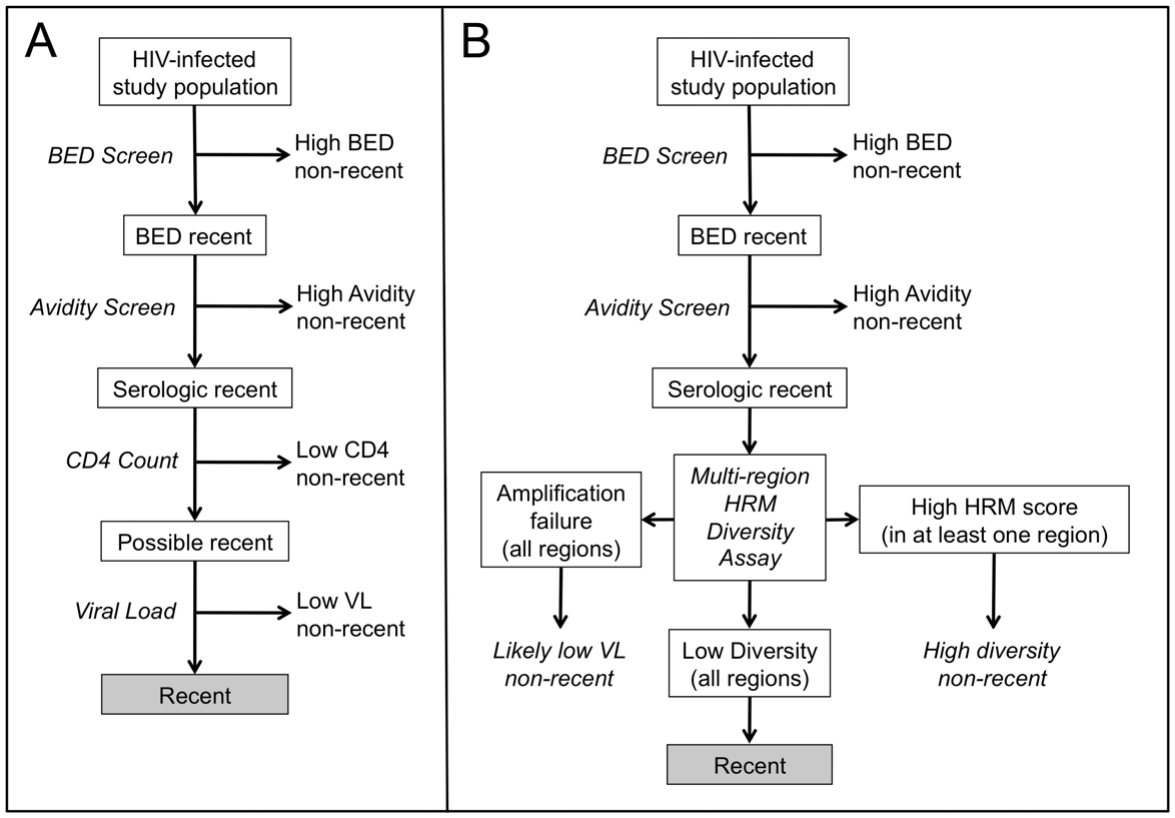
Use of the HRM diversity assay as part of a multi-assay algorithm for HIV incidence determination. Panel A shows one example of a multi-assay algorithm developed for HIV incidence determination. In this algorithm, samples from HIV-infected individuals are first tested using the BED-CEIA assay, using a high assay cutoff to indicate non-recent HIV infection (BED screen). Samples that are below the BED screen cutoff (BED recent samples) are then tested using a second serologic assay, such as one based on antibody avidity (avidity screen). Samples that are below the cutoff for the second serologic assay are considered to be “serologic recent” samples. Samples with low CD4 cell count test results are then excluded as non-recent (note that CD4 cell count test results are usually obtained for all HIV-infected individuals at the time of sample collection). Finally, samples that are not excluded based on CD4 cell count are tested using a viral load assay, and samples with low viral loads are excluded as non-recent. The remaining samples are characterized as recent for the purpose of estimating HIV incidence. Panel B shows an alternative multi-assay algorithm that incorporates the HRM diversity assay. In this algorithm, samples that are characterized as serologic recent based on two assays (BED screen and an avidity screen) are tested with a multi-region HRM diversity assay. Samples that have a high HRM score in at least one of the regions tested are excluded as non-recent. Samples that fail to amplify in all regions tested are also excluded as non-recent, based on the assumption that they have low viral loads; this could be confirmed with a viral load assay. Samples that have low HRM scores in all regions tested are characterized as recent for the purpose of estimating HIV incidence.

The HRM diversity assay provides data that is likely to be independent of data from serologic incidence tests. Therefore, use of the HRM diversity assay in combination with serologic testing is likely to improve the precision of multi-assay algorithms for HIV incidence, lowering misclassification rates. Figure 5A shows an example of an existing multi-assay algorithm that combines four assays for HIV incidence determination: a BED screening assay and an avidity screening assay (using a high cut-off for recent HIV infection for both assays), CD4 cell count, and viral load [23], [37], [38]. While CD4 cell count is a useful biomarker for reducing misclassification, inclusion of CD4 cell count data in incidence algorithms presents certain logistical challenges. First, because CD4 cell counts must be obtained in real-time (before other HIV incidence testing has been performed), CD4 cell count testing must be performed for all HIV-infected individuals evaluated, rather than the smaller subset who appear to be recently infected based on serologic testing. Second, many valuable sample sets from clinical trials and surveillance studies include only stored serum or plasma. Unless CD4 cell counts were obtained at the time of sample collection, it is not possible to assess incidence in those sample sets using an algorithm that includes CD4 cell count data.

We are exploring whether inclusion of the HRM diversity assay as part of a multi-assay algorithm will eliminate the need for CD4 cell count data, allowing all of the testing to be performed using a single plasma or serum sample. Figure 5B shows a possible alternative multi-assay algorithm that incorporates the HRM diversity assay. To reduce the cost and effort needed for analysis, it would be most effective to screen samples for recent infection using serologic HIV incidence assays (e.g., BED and avidity screens). The subset of samples that are classified as “recent” based on serologic testing could then be tested using the HRM diversity assay to improve the precision of HIV incidence estimates. With this analytic plan, the number of samples that would require HRM diversity analysis would be relatively small. Therefore, the cost of the HRM diversity assay would not greatly impact the overall cost of the incidence assessment. Our preliminary data suggest that HIV from recently-infected individuals usually lacks diversity across the HIV genome, while HIV from individuals with non-recent infection is genetically diverse in one or more genomic regions. Studies are underway to identify genomic regions and assay cutoffs in each region that optimally discriminate between recent and non-recent infection. Once those parameters are set, it might be possible to use a simple approach in which samples are characterized as non-recent if they have a high HRM score in at least one of several regions tested.

In summary, this study provides proof of principle that HIV diversity can be used as a biomarker to distinguish between adults with recent vs. non-recent HIV infection. Further studies are needed to evaluate the performance of multi-assay algorithms for HIV incidence determination that include the HRM diversity assay.
